# Feasibility of Colocating a Nutrition Education Program into a Medical Clinic Setting to Facilitate Pediatric Obesity Prevention

**DOI:** 10.1177/21501327211009695

**Published:** 2021-04-13

**Authors:** Mical K. Shilts, L. Karina Diaz Rios, Katherine H. Panarella, Dennis M. Styne, Louise L. Lanoue, Christiana M. Drake, Lenna Ontai, Marilyn S. Townsend

**Affiliations:** 1 California State University, Sacramento, Sacramento, CA, USA; 2 University of California, Merced, CA, USA; 3 University of California Agriculture and Natural Resources, Davis, CA, USA; 4 University of California, Davis Medical Center, Sacramento, CA, USA; 5 University of California, Davis, CA, USA

**Keywords:** nutrition education, community health, children, health promotion, mixed methods, obesity, pediatrics, underserved communities

## Abstract

**Purpose:**

Within a medical clinic environment, pediatric obesity prevention education for families faces challenges. Existing long-term government-funded nutrition education programs have the expertise and staff to deliver. The purpose is to determine feasibility of colocating the Expanded Food and Nutrition Education Program (EFNEP) into a medical clinic setting to support pediatric obesity prevention.

**Methods:**

Physicians from a large university teaching and research hospital (n = 73) and 4 small Medicaid-serving community clinics (n = 18) in the same geographic area in northern California were recruited and trained in the patient-referral protocol for a primary prevention intervention provided by EFNEP. The 8-week intervention deployed in the medical clinics, included general nutrition, physical activity and parenting topics anchored with guided goal setting and motivational modeling. Referral, enrollment, and attendance data were collected for 2 years. Parent and physician feasibility surveys, parent interviews and parent risk assessment tools were administered. Paired-sample t-test analysis was conducted.

**Results:**

Twenty intervention series with parents of patients (n = 106) were conducted at 5 clinics. Physicians (n = 92) generated 686 referrals. Every 6 referrals generated 1 enrolled parent. Physicians (91%, n = 34) reported the intervention as useful to families. Parents (n = 82) reported improved child behaviors for sleep, screen time, physical activity, and food and beverage offerings (*P* < .0001) and at family mealtime (*P* < .001). Focus group interviews (n = 26) with 65 participants indicated that parents (97%) reacted positively to participating in the intervention with about a third indicating the classes were relevant to their needs.

**Conclusion:**

The intervention is a feasible strategy for the 5 medical clinics. Physicians referred and parents enrolled in the intervention with both physicians and parents indicating positive benefits. Feasibility is contingent upon physician awareness of the intervention and motivation to refer patients and additional EFNEP and clinic staff time to enroll and keep parents engaged.

## Introduction

Obesity continues to disproportionately impact low-income, ethnically diverse populations with 19% of low-income youth and 39% of adults (≤130% Federal Poverty Level [FPL]) categorized as obese in comparison to higher income (>350% FPL) youth 11% and 31% adults.^1,2^ Obesity is a serious public health concern with trends projecting an estimated 6 to 8.5 million more cases of diabetes and 5.7 to 7.3 million more cases of heart disease due to obesity by 2030. The estimated increase in medical costs due to obesity is $48 to 66 billion/year.^3^ Physicians are motivated to address pediatric obesity if time efficient strategies are available.^4,5^ Unfortunately, limited nutrition training in medical school and time constraints during clinic visits create a void in patient care for the management of pediatric obesity.^6-9^ A potential solution is to establish collaborations between pediatric medical clinics and existing government-funded nutrition education programs with expertise in delivering behaviorally-focused culturally sensitive interventions.^10,11^

A national nutrition education program, The Expanded Food and Nutrition Education Program (EFNEP), funded through United States Department of Agriculture (USDA) National Institute of Food and Agriculture and operated through Cooperative Extension has been serving low-income families since 1969.^12^ EFNEP has a presence in all 50 states and 6 territories, thus has the potential to impact obesity prevalence among low income families.^13^ EFNEP has been shown to produce long-term positive impact on food and nutrition-related behaviors^14^ and overall quality of life of its families.^15^ EFNEP in California (CA) delivers group-based nutrition education interventions at local community agencies like Head Start, public schools, food banks, and community centers. Although EFNEP is excellent at building partnerships with existing programs serving low-income families, medical clinics are yet to be targeted as partners for enrollment in most of the country, including California.^16^

The Cooperative Extension National Framework for Health and Wellness recommends Cooperative Extension Service (CES) explore partnering with healthcare professionals for health promotion and disease prevention by providing community education.^17,18^ One EFNEP program in the Eastern United States has reported successful collaboration with 3 medical clinics to enroll families in nutrition education classes delivered at the medical clinic.^19^ Recently, the *Rx for Health Referral Toolkit* was developed and pilot tested to link medical clinic patients to CES programming like EFNEP. In this model, patients are referred to CES by medical clinic staff with interested patients attending existing classes in the community setting.^11^ There appears to be momentum for CES programs like EFNEP to partner with medical clinics.

EFNEP educators by statute do not deliver medical nutrition therapy for disease treatment but rather focus on general nutrition, obesity prevention, food safety and food security. Clinics with Medicaid patients would be a suitable partner for EFNEP because both target low-income families. EFNEP colocated in a medical clinic setting has potential beneficial outcomes to patients, EFNEP, and medical clinics. EFNEP could benefit from physician referrals to expand its reach to otherwise underserved clientele. Physicians and medical clinics could benefit from patients receiving nutrition education. Patients would receive free family-focused, nutrition education in the convenience of their neighborhood medical clinic. Hodge^20^ described the potential opportunities for nutrition integration in medical clinic settings and receiving advice from different health professionals as an important way to improve population health; but evidence on the feasibility and potential impact of this type of approach is lacking.

The objective of this study is to determine feasibility of colocating an EFNEP intervention in a medical clinic setting to support pediatric obesity prevention. This study specifically addresses the following aspects of feasibility: demand, acceptability, and limited efficacy testing.^21^

## Methods

### Procedure

**Figure 1.**
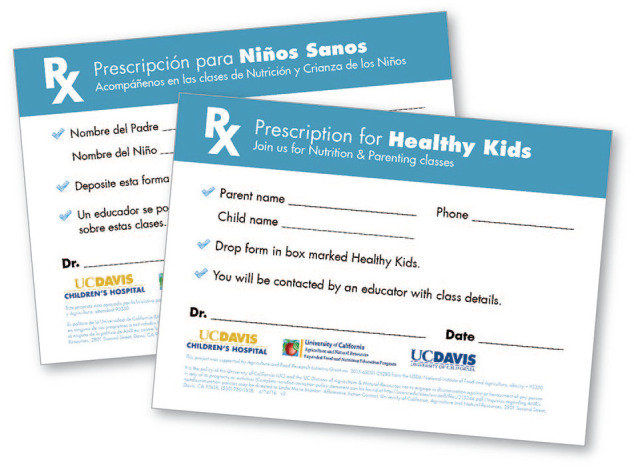
Intervention referral sheet in English and Spanish.

Physicians from a large university teaching and research hospital (n = 73) and 4 small Medicaid serving community clinics (n = 18) in the same geographic area in northern California were recruited and trained in the patient-referral protocol for an 8-week primary prevention intervention provided by EFNEP. Pediatric obesity risk factors, assessment and treatment strategies were also included in the training. Referral sheets (Figure 1) were developed with input from medical staff and printed in pads of 50, simulating the dimensions of medication prescription pads. Referral sheets and recruitment materials were deployed at 8 locations within the hospital and clinics. All materials were created in English and Spanish.

Targeted intervention participants were low-income, English and Spanish speaking parents or primary caregivers, that is, grandparents or foster parents, of children (<18 years) who would benefit from attending an intervention focused on reducing obesogenic behaviors under control of the family. The physician completed the referral during the medical clinic visit for the child and the parent provided contact information. Referral sheets were placed in designated, locked referral boxes at each clinic by either the parent or physician. Referral slips were collected by our research staff weekly and telephone calls for parent enrollment were made 2 to 3 weeks before the beginning of an intervention series. Research staff also sent reminder calls and text messages to the enrolled parents the day before each class session.

Intervention educators (n = 4) were from the University of California Cooperative Extension (UCCE) Expanded Food and Nutrition Education Program (EFNEP) in Northern California with an average of 8.75 years of service. EFNEP educators have cultural competence and language skills to best serve the targeted populations. They receive nutrition training by Cooperative Extension Nutrition Specialists based at land grant universities. Two of the 4 educators were bi-lingual and taught the Spanish language intervention series. Educators completed the Human Research training and were individually trained by the research staff on the study protocol. Educators completed post-session logs to document process-related information including attendance, special circumstances or lesson alterations and barriers to delivery. Data collection, including pre and post intervention assessment tools, was supervised by research staff. Parents received $20 to 25 grant funded stipends for completing the assessment tools and attending the intervention sessions to assist with transportation, parking, and childcare costs and $10 for participating in a 1-hour post-intervention interview. Top referring residents at the teaching hospital were given $10 coffee shop gift cards. The study protocol was approved by the Institutional Review Board’s at University of California, Davis and California State University, Sacramento.

### Intervention

The CA EFNEP Director and staff, UC Davis Medical Center Pediatric endocrinologist and nursing staff, UC Davis and UC Merced Nutrition Specialists, and UC Davis Human Development Specialist participated in 3 planning meetings. Referral protocol was developed and intervention content designed with focus on pediatric obesity prevention while fulfilling the needs of the medical clinic and EFNEP guidelines. The resulting intervention included 5 components: traditional EFNEP Eating Smart, Being Active (ESBA) curriculum; guided goal setting; motivational modeling; MyHealthyPlate; and parenting and child feeding practices. Guided goal setting^22,23^ based on [Blinded] Healthy Kids and My Child and Mealtime pediatric obesity risk assessment tools^24,25^ replaced the self-set goal setting in the traditional EFNEP Eating Smart Being Active curriculum^26^ to enhance tailoring and effective goal setting strategies related to pediatric obesity risk. Parenting and child feeding topics from the Healthy, Happy Families Curriculum^27^ and Motivational Modeling^28,29^ were also added to further address determinants of pediatric obesity. MyHealthyPlate activities were included to address recommended food group proportions and child appropriate portion sizes relevant to low-income audiences.^30^ Additional tailoring was conducted to customize content for a Spanish speaking audience. The tailoring included culturally relevant recipes and all handouts, assessment tools, and promotional materials were translated and contained culturally appropriate images. The final intervention included 8 weekly 1.5 hour sessions (Supplemental Table S1).

### Measures and Data Analysis

The work of Bowen et al^21^ guided a mixed methods approach using quantitative and qualitative methods to explore 3 areas of feasibility: demand, acceptability, and limited-efficacy testing. Demand for the EFNEP intervention was assessed by tracking physician referrals, parent enrollment calls/text messages, and intervention attendance. Acceptability or “how the intended individual recipients react to the intervention” was measured via self-administered parent and physician surveys and parent focus group interviews. A 1-group pre and post intervention assessment to test behavior change was used for limited-efficacy testing.

#### Intervention demand

Research staff collected physician referral sheets weekly from clinic sites. Date referred, clinic name, parent contact information, and physician name were documented in a spreadsheet. Enrollment calls, text messages, enrollment, and class attendance were also tracked on the spreadsheet. Descriptive statistics included frequencies and percentages and Chi-square tests were performed using R version 3.6.3^31^ to compare enrollment and attendance data between the English and Spanish language intervention series.

#### Acceptability

Parents completed an 8-item survey in English or Spanish assessing acceptability and feasibility of the EFNEP intervention in a medical clinic setting. Survey content covered motivators for class attendance, acceptability of intervention strategies, and barriers to attendance with 4-response Likert scales (4 = very important, 1 = not important and 4 = very important, 1 = not important) and multiple-choice answer options (Supplemental Table S2). The survey had a Flesch-Kincaid readability index of grade 4. Physicians at participating clinics were invited to complete a 13-item feasibility survey online. Survey content included referral activity, referral barriers, perceived EFNEP intervention usefulness, and suggestions for improvement with 5-point Likert scales (5 = very useful, 1 = not useful), multiple choice, and open-ended response options (Supplemental Table S3). Descriptive statistics included means, frequencies and percentages for both surveys.

Parents were invited to attend focus group interviews conducted in English and Spanish to assess the relevance and appeal of the EFNEP intervention in a medical clinic setting. A semi-structured question guide was created with input from all members of the research team to explore parents experience participating in the intervention (Supplemental Table S4). A bilingual researcher with expertise on qualitative methods conducted the interviews while trained research staff took notes. Interviews were audio-recorded and transcribed verbatim by trained bilingual and bicultural research staff. Data was compiled and organized in Microsoft Excel for content analysis.^32^ A codebook was created by a trained analyst and an experienced researcher using a priory categorization and refined via emerging coding.^33^ Data were then independently coded by 2 trained bilingual and bicultural analysts who conferred with a third analyst to resolve disagreements in code assignment. Codes were quantified in a pivot table to determine their density and representativeness across participants.

#### Limited-efficacy

Parents completed the 19-item University of California Cooperative Extension Healthy Kids (HK; Supplemental Table S5) assessment tool, the 27-item companion tool, My Child and Meal Time (MCMT; Supplemental Table S6), and the 15-item EFNEP Behavior Checklist in English or Spanish pre and post intervention.^24,25,34,35^ The HK and MCMT tools were developed to measure specific modifiable behaviors associated with pediatric obesity in low-income populations with HK focusing on dietary, physical activity, screen time and sleep and MCMT focusing on parenting and family mealtime behaviors.^36,37^ The California pictorial version of the EFNEP checklist focuses on parent behaviors related to food safety, meal planning, food insecurity, sugar-sweetened beverages, fast-food, screen time, breakfast, and the food label using text and visuals for limited literacy learners.^35^ The Flesch-Kincaid readability index of HK was grades 1 to 2, MCMT was 2 to 3, and EFNEP checklist 1 to 2, making them suitable for the low-income parents with literacy issues in this study. Reliability and validity has been demonstrated for the HK and MCMT measures.^38-42^ HK and MCMT instruction guides were used to ensure consistent administration.^43,44^ HK parent responses were coded using 5 response options or 5 points per item. The most healthful response was given the higher score (5) and the minimum of 1 point to the least healthful response. The HK items were summed into a total score. Similarly, MCMT responses were coded using 4 response options for a maximum of 4 points per item and summed into 2 subscales: child-centered and parent-centered behaviors. The EFNEP behavior checklist responses were coded using 5 response options per item for a maximum of 5 points per item. The most healthful response was given the higher score (5) and the minimum of 1 point to the least healthful response. The 15 items were summed into a total score. A paired sample *t*-test was performed using SAS/STAT^®^ software version 9.4(SAS Institute, Cary NC) and R version 3.6.3^31^ to compare the total score and scales before and after the intervention. Significance level was set at *P* ≤ .05.

## Results

### Intervention Demand

**Figure 2.**
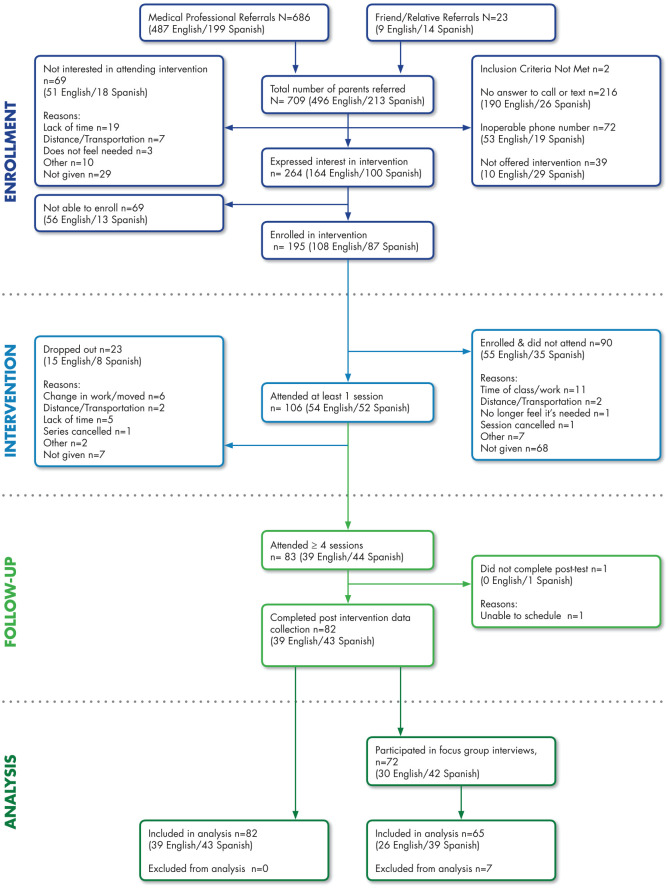
Medical clinic referral and intervention enrollment flowchart.

Physicians (n = 92) from 1 large university teaching and research hospital and 4 small Medicaid serving medical clinics generated 686 patient referrals (Figure 2). Spanish-speaking parents represented 29% of the referrals. Physicians averaged 8 referrals while 1 physician generated more than 25% of the referrals. More than one-third (n = 264; 38% Spanish-speaking) of these patients expressed interest in attending the EFNEP intervention with 195 verbally agreeing to be enrolled in a specific intervention series. Parents who attended at least 1 session (n = 106; 52% Spanish-speaking) were mostly female (95%), Hispanic (67.9%) and low-income (81% participated in an assistance program; Table 1). Approximately 6 referrals were needed to get 1 parent enrolled in an EFNEP intervention series. An average of 15 phone calls and texts were made by staff to enroll and remind each parent to come to each of the 8 sessions.

**Table 1.** Demographics of Parent’s Attending the EFNEP Nutrition Intervention in a Medical Clinic Setting (N = 106).

Demographics	n (%)
Parent gender (female)	95 (90)
Parent age (mean [SD])	37.4 (8.2)
Income per month	
<$2000	54 (51.9)
$2001-3500	35 (33.7)
>$3501	15 (14.4)
Percent participating in an assistance program	86 (81.1)
Parent ethnicity (Hispanic)	72 (67.9)
Parent race	
African American	16 (15.1)
Asian	3 (2.8)
White/Caucasian	27 (25.5)
Multiple races selected	11 (10.4)
No response	49 (46.2)
Child gender (female)	61 (59.2)
Child age, years (mean [SD])	7.5 (4.2)

Twenty intervention series were conducted over a 2.5 year period in English (n = 12) or Spanish (n = 8) at 5 medical clinics by 4 EFNEP educators. Recruitment was enhanced by encouraging referred parents to invite a family member or friend to attend which resulted in an additional 23 parent referrals. Most enrolled parents (78%) completed 4 or more sessions with many bringing their children, friends or family members to some of the intervention sessions. A greater proportion of Spanish-speaking parents expressed interest in the intervention (*P* = .0004) and completed 4 or more sessions compared to English-speaking parents (*P* = .0006; Table 2).

**Table 2.** Intervention Referral, Enrollment and Attendance by English and Spanish Speaking Parents.

	Total	English n; %	Spanish n; %	*P* value and χ^2^ statistic*
Clinic referrals	709	496; 70	213; 30	—
				
Expressed interest	264	164; 33	100; 47	12.29
Not interested	445	332; 67	112; 53	.0004
				
Attended ≥4 intervention sessions	83	39; 24	44; 44	11.78
Attended <4 intervention sessions who expressed interest	181	125; 76	56; 56	.0006

* Chi-square test.

### Acceptability

Parent (n = 82) acceptability of the EFNEP intervention was demonstrated with most parents rating intervention content favorably. Parents “liked very much” the nutrition content (94%), goal setting activities (88%) food tasting (93%) and parenting topics (90%). Most parents (73%) reported classes in their medical clinic as important or very important reason for coming to the classes. Physician referral was identified as an “important” or “very important” reason for enrolling (83%). A parent commented that they scheduled a physician appointment before the EFNEP class demonstrating convenience. Less than half of the parents (41%) said the stipend was very important. During interviews, parents mentioned the stipend was appreciated but they would come without it. A majority (93%) of parents reported other parents they know would attend these classes. When asked about potential barriers to attendance, 52% said childcare could be an issue.

Focus group interviews (n = 26) with 65 participants were conducted immediately after the last session of the intervention. Interviewees represented 18 of the 20 series offered. Interviews were conducted in Spanish (n = 14), in English (n = 11), and in both languages simultaneously (n = 1). On 6 occasions, more than 1 interview was conducted for the same series to accommodate parents’ availability.

Most of the parents (97%) interviewed said they enjoyed participating in the intervention, with about a third indicating the classes were relevant to their needs. (Table 3. Representative Quotes from Parent Interviews):

“Every information that was given to me [is] something that will enrich my family, my health, my home. *(Cada información que se me dio [es] algo que va a enriquecer a mi familia, mi salud, mi cas).”* (Particpant (P)33, Focus Group (G)13)

**Table 3.** Representative Quotes from Parent Interviews.

Category, code	Representative quotes
Appeal	
Positive n = 375 R = 97%	I liked it real good. It was nice. I learned a lot. (P10, G4). It was a very nice [experience]. I learned a lot . . . it was something wonderful. (P11, G5) * Para mi fue [una experiencia] muy bonita. Aprendí muchísimo . . . fue algo maravilloso*. I'm really happy with it. I’m glad I took this class—it helped me out a lot. (P17, G6, L590) [The classes were] very informative and enriched my life. (P25, G10) I like the class. I really enjoy, you know, having the time to discuss and learn. (P41, G16) [The classes] were phenomenal. It was the best thing that could have happened to me, because I learned a lot. (P52, G21) * [Las clases] fueron fenomenales. Fue lo mejor que me pudo haber pasado, porque aprendí mucho.* It was a good, good experience. (P47, G19) I love the class. (P55, G22) It was very beautiful and very perfect for me . . . I don't know how to thank you for the change I have made. (P58, G23) * A mí se me hizo bien bonito y bien perfecto . . . No sé cómo agradecerles el cambio que he hecho* I thought it was wonderful—very helpful and informative. (P60, G25)
Relevance n = 48 R = 31%	I have taken other classes like these; but these were, like, more at ease. (P12, G5) * He tomado mas clases de estas; pero estas eran como mas en confianza. . .* [At the beginning], of course one doesn't know much. But what one feels after finishing this is. . .like a lot of power. (P28, G11) * [Al principio], claro que uno no sabe mucho, pero lo que siente uno después de terminar esto es. . .como mucho poder.* I liked the variety of information, [it helps to] learn better and be able to apply it to our daily lives, with my children. (P64, G26) * Me gusto la variedad de información, [ayuda a] aprender mejor y poderlo aplicar a la vida diaria de nosotros, con mis hijos.*
Usefulness	
Component – goal setting n = 70 R = 63%	The goals [were very helpful]. I had a goal to go for a walk every day with my children. (P6, G3) * Las metas [fueron muy útiles]. Yo tuve una meta en salir a caminar todos los días con mis niños.* The goal setting—I liked that part. I think more how set a goal and have a certain amount of days to do it. (P10, G4) [The goals] held me accountable. (P21, G8) I leave with great motivation and goals to work on. (P30, G12) * Me voy con una gran motivación y metas para seguir.* You set up the goal. You set up to follow the goal—to eat correctly and healthy . . . I felt it as if it was a seed that I had to harvest. (P39, G16) * Yo la sentí como si era una semilla que la tuve que cosechar.* The goal setting part was useful—it was very useful (P47, G19) The goals were remarkable to me. They helped me spend more time with my family and especially my son, because sometimes we don't have time. And they help me grow that way. (P54, G21) * Los goles [sic] fueron brillantes para mí. Me ayudaron a pasar más tiempo con mi familia y especialmente con mi hijo, porque sé que a veces no tenemos tiempo. Y me ayudó a crecer de esa manera.* One of the most useful [parts] was the goal setting. . . . not only just setting that goal but the accountability of talking about [it] ‘did we reach our goal?’ How we could have reached our goal better and just the support from everyone else. (P57, G22) [The goals] kept you on your toes. What’s that word? Accountable. (P61, G25)
Content – recipes & food choices n = 121 R = 63%	The very easy recipes she gave us. And they are simple, with ingredients we often have around and don’t use. And they are tasty. (P17, G6) * Las recetas tan fáciles que nos dio. Y son simples, con ingredientes que a veces tenemos ahí guardados y no los usamos. Y salen ricas.* It motivated me so to . . . include vegetables in my diet, because I rarely eat them. And now we are eating more as a family. (P18, G7) * Me motivo mucho a . . . incluir los vegetales en mi alimentación, porque yo casi no como. Y ahorita ya estamos comiendo más la familia.* They even gave us food recipes and tips. (P27, G11) * Hasta nos dieron recetas de comidas y también los consejos.* I liked getting the recipes each week—opening my ideas of different kinds of snacks I can offer or different ways of doing things. (P42, G17) I got to learn more recipes, so that was great. (P59, G24) It was nice to learn new recipes—different ways to make food. I enjoyed the food, even the tuna was great [laughs]. (P61, G25)
Content – food labels n = 64 R = 48%	I learned about how to shop in stores. Something I never did, now I stop and see the labels. (P14, G6) * Aprendí sobre como comprar en las tiendas. Algo que yo no nunca hacia, ahora me pongo a ver las etiquetas.* Previously, I didn’t read the labels — it just said “100%, healthy” and I bought it. But I found out how to read them now, I know what the deal is—I know if it’s a healthy product, or sugary, or salty. I know how to differentiate now. (P35, G13) * Anteriormente yo no leía las etiquetas—simplemente decía que ‘100%, sano’ y yo lo compraba. Pero ya me enteré de cómo se debe de leer, ya se dé que se trata—ya sé si es un producto saludable o azucarado o salado. Ya sé diferenciar.* For me, what was helpful was learning to read food labels. (P50, G20) * Para mí, lo que fue útil fue aprender a leer las etiquetas de los alimentos.* I enjoyed learning how to read labels correctly. (P62, G25)
Knowledge gain n = 80 R = 62%	[The classes] brought things to my attention that I didn't realize. (P9, G4) From these classes we learned many new things. (P14, G6) * De estas clases aprendimos muchas cosas nuevas.* We learned a lot. It covers everything. It does not just cover . . . ‘you have to eat vegetables, you have to eat fruits.’ (P18, G7) * Fue bastante lo que aprendimos. Abarca todo. No nomas abarca . . . ‘tienes que comer verduras, tienes que comer frutas.’* We learned many things that we did not know. (P48, G20) * Aprendimos muchas cosas que no sabíamos.*
Behavior change n = 49 R = 43%	I got really good information in things that have definitely helped make some changes that I wasn’t expecting (P42, G17) I have been practicing at home and have seen results from what I have learned here. (P50, G20) * He estado practicando en casa y he visto resultados de lo que he aprendido aquí.*

Abbreviations: n, code frequency; R, representativeness = (number of participants using code * 100)/total number of participants.

Most participants (97%) also found the classes useful:

“Pretty much everything that was taught in this class was useful. I can’t really pick one particular thing because everything that was talked about was always important. It was a tool that you could use no matter what the subject was.” (P56, G22)

The intervention component most commonly mentioned as useful was the guided goal setting. The topics most frequently alluded to as useful were those meant to help improve their food choices, including provided recipes, food demonstrations, and reading food labels activities. Most interviewed parents also said they learned new information or skills, with a number of them (43%) stating they either intend to apply or are already applying what they learned at home:

“We have changed. We have had a radical change. It has helped us a lot. Is like our eyes were opened and now we are going to put it into practice. *(Pues hemos cambiado. Hemos tenido un cambio radical. Nos ha ayudado mucho. Haz de cuenta que nos abrieron los ojos y ahora lo vamos a poner en práctica).” (P29, G12)*

According to parents’ feedback, elements of the intervention elicited an effective learning environment, including educators’ cultural competence and rapport, family-friendly environment, and opportunities for social interaction. Many parents praised the educator’s ability to connect with them, to make the content relatable and clear:

“[The educator] had the disposition to approach us and inspire trust. Sometimes, one goes to the doctor and the providers don’t inspire the trust [the educator] does. *([La maestra] tenía como carácter para tratarnos y darnos la confianza. A veces uno va a los doctores y las personas que lo atienden no tienen como esa confianza que da [la maestra])*.” (P12, G8)

Thirty-four physicians (82% residents) completed the online feasibility survey assessing acceptability of the EFNEP intervention in their medical clinic. Almost all physicians (97%) indicated that physicians at other clinics would participate in referring patients if given the opportunity. A Pediatric Resident stated, *“I think it's a great opportunity to get patients nutritional advice! I would expect other doctors to be excited about this opportunity as well.”*

More than two-thirds of the participating physicians referred patients to the EFNEP intervention and more than half made (64%) made 4 or more referrals. Those that did not make referrals stated that they were not aware of the opportunity (46%). When asked what would help them make referrals, physicians suggested the need for more reminders and emails, flyers about the program, dates of when classes/sessions are starting, and placing referral box in more visible location. Most physicians found value in the EFNEP nutrition intervention for parent/patients (91%) and physicians (88%). *“I had 1 patient come back to clinic and be very proud of the modifications they had made at home. She was feeling happier and proud of herself. The mother had also made many improvements in her life.”* Physicians were asked to provide suggestions to improve training and transmittal of information about the intervention. Some physicians suggested the need for a feedback mechanism for referral outcomes including when referred patients were contacted, which referred patients attended the intervention, and potential positive impacts.

### Limited-Efficacy Testing

#### Pre and post intervention survey results

Parents who completed at least 4 intervention sessions and the assessment tools (19-item HK tool, 27-item MCMT, and 15-item EFNEP Checklist), pre and post intervention were included in the limited-efficacy testing (n = 82). There was a significant difference between the HK 19-item pediatric obesity risk assessment tool pretest scores and the posttest scores (*P* < .0001) indicating families improved child behaviors for sleep, screen time, physical activity, and food and beverage offerings (Table 4). Parents also reported improvement in family mealtime behaviors via MCMT assessment tool with fewer parent-centered behaviors (i.e., using rewards or pressure to encourage eating; *P* < .001) and more child-centered behaviors (i.e., making food fun and letting child serve herself; *P* < .0001). Lastly, the EFNEP Checklist results indicated parents made significant improvements in food resource management, food safety, nutrition practices, and screen time behaviors after the intervention (*P* < .0001; Table 4).

**Table 4.** Family-Based Dietary and Activity Environment and Parent Food-Related Behaviors Assessment Tool Scores Before and After the EFNEP Intervention (n = 82).

Scale	Pre *M* (SD)	Post *M* (SD)	Difference *M* (SD)	*t*	*P* value*
Healthy kids					
19-items including behaviors associated with child BMI: bedtime, fruit and vegetables, activity, snacking, and sweetened beverages	68.31 (8.69)	72.43 (7.92)	4.12 (6.78)	5.40	<.0001
My child at mealtime					
27-items including family meal time behaviors					
Child-centered scale	2.74 (0.46)	2.99 (0.45)	.25 (0.48)	4.81	<.0001
Parent-centered scale	1.69 (0.41)	1.56 (0.43)	−.13 (0.34)	−3.38	<.001
EFNEP checklist					
15-items including behaviors and knowledge on food resource management, food safety, nutrition practices and screen time behaviors.	54.68 (5.42)	59.26 (5.98)	4.58 (0.21)	−6.89	<.0001

* Paired sample *t* test.

## Discussion

Results from this study indicate this is a feasible strategy as physicians referred patients and patient’s parents enrolled and completed the intervention both indicating positive benefits. The parents specified the intervention was relevant to their needs. The nutrition education program benefited from physician referrals and use of clinic site for lessons. The physicians and medical clinic benefited from the patient’s parent receiving behaviorally-focused, pediatric obesity prevention intervention, an intervention they would have neither the language skills, nutrition expertise nor time to deliver.^6-9^ Such an intervention is not eligible for Medicaid reimbursement at the hospital or clinic. A partnership with an established, federally-funded nutrition education program, such as EFNEP, represents a cost-effective solution to a prevailing need. However, feasibility is contingent upon physician motivation to refer patients and additional medical clinic or nutrition program staff time to contact referred patients for enrollment. It took 6 physician referrals and additional EFNEP staff time (~45 minutes) for every parent enrolled across an 8 week series.

Two factors appeared to enhance feasibility in this research: demand and acceptability as described by Bowen et al.^21^ In this study, demand is a function of physician characteristics while acceptability is based on patient demographic characteristics. One physician referred over 25% of all patients to the intervention. This physician was different in some ways compared to other (n = 80) referring physicians. First, she was not a resident at a large teaching hospital; instead, she was a practicing pediatrician at a small community clinic. Working at a small clinic most likely allowed her to establish closer relationships with her patients compared to a large teaching hospital environment. The physician also initiated the contact with the research team indicating desire for nutrition education for her patients as opposed to residents being offered to take part in this project. On the other hand, the partnership with the large teaching hospital allowed more physician residents to be involved compared to the small clinics with fewer physicians. The physician residents did refer a majority of the patients in this study. However, physician resident turnover after 1 to 3 years may limit the necessary continuity in the relationship with patient families for intervention acceptance. The annual turnover also required additional time to inform and train new residents on the intervention and referral process by EFNEP staff with each new cohort.

The second factor enhancing feasibility in this study was acceptability. Intervention classes delivered in Spanish had more participants per class and a higher retention rates compared to the classes delivered in English (Table 3). Only 30% of the referrals were for Spanish-speaking parents yet they accounted for 53% of parents who completed the intervention. Similarly in the NET-Works study, Hispanic parents participated in more intervention sessions and were the only group to have a BMI reduction after the 3-year intervention that integrated home visiting, community-based parenting classes, primary care provider interactions, and neighborhood connection strategies.^45,46^ Offering classes in languages that match the need of the community is a tenet of EFNEP as they hire educators who are linguistically and culturally competent and teach in these communities.^12^ Culturally competent educators and relevant content and delivery have been identified as key to successfully reaching Latino/Hispanic groups for community nutrition research.^47^ In addition to trusted educators, other recommended approaches were employed to ensure participation and retention of both, Spanish- and English-speaking parents. These included (1) partnering with trusted community clinics for referral, recruitment, and delivery, and (2) allowing family members to come to the classes.

### Limited-Efficacy Testing

Parents reported changes in the predicted direction for better outcomes in all targeted pediatric obesity prevention behaviors measured. The intervention strategies were evidenced-based, theory-driven and previously shown to be feasible and/or effective.^23,26,27,29,30^ Focus group interviews revealed that guided goal setting was the most commonly mentioned useful intervention component and parent feasibility survey results corroborated this. Since study scope was limited by convenience sampling and no control group, the results do support further investigation to test efficacy and effectiveness.

### Sustainability

It is worth noting that all of the medical centers participating in the feasibility study continued participation after the conclusion of the study. One medical clinic provider integrated the referral, enrollment and feedback process into the electronic medical record (EMR) system where the physician electronically refers the patient to the EFNEP intervention during the clinic appointment. The clinic health education department is then notified of the referral and like other types of physician referrals, patients are contacted by clinic staff to query patient interest in attending the nutrition intervention. If the patient is interested, he/she is enrolled in the next series of classes in their preferred language. Follow-up calls are made by the clinic for the first 3 sessions after which the EFNEP educator continues the reminder calls. This enhanced partnership between this clinic and EFNEP has improved the sustainability and reach of the program. The EFNEP educator is able to focus on his/her expertise in culturally competent nutrition education. The medical clinic is more involved in the enrollment and has an internal mechanism for following up and documenting intervention participation. Future research may explore the stability of this model at this clinical setting and its impact on nutrition outcomes of participating families. Similarly, Maryland EFNEP has also collaborated with medical clinics serving low-income families.^19^ Importantly, the referral process was embedded in the patient electronic medical record (EMR) and recruitment was coordinated by a clinic referral specialist, not EFNEP staff. This process resulted in 31% completion rate (completion rate = # of parents who completed intervention/# of referred parents). Our study had a lower completion rate of 12% overall with 8% for English-speaking parent classes and 21% for Spanish-speaking parent classes. Benefits from a shared workload between the medical clinic and the EFNEP educator for recruitment, enrollment, and follow-up appear to be important. Other researchers conducted a study where a family nutrition and physical activity risk assessment was delivered in the medical clinic during the well-child visit and data was integrated into the electronic medical record to deliver preventative counseling. The study found that integrating the risk assessment into the clinic visit/EMR/providers’ workflow is feasible but clinician utilization and counseling practices should be continuously evaluated.^48^ This could also serve as a point for referral to community intervention like those provided by EFNEP.

With many (71%) of US medical schools failing to provide the recommended minimum 25 hours of nutrition education to physician students,^49^ it is no surprise that less than 15% of resident physicians reported feeling adequately prepared to deliver nutrition counseling, although most agreed that nutrition assessment and education were important.^50^ One study found pediatricians (>90%) were interested in childhood obesity prevention.^5^ Brown and Perrin^6^ specified that collocating nutrition support personnel in the clinic and working with community resources such as Cooperative Extension Service programs including EFNEP would be important facilitators. Pediatricians (82%) reported that many patients are not able to pay for uncovered services such as nutrition education.^5^ A benefit of medical providers collaborating with an existing externally-funded program like EFNEP, is that the payments for services is a non-issue. Our study adds to the initial groundwork in detailing a model to facilitate this type of collaboration.^11,19^ Because EFNEP operates in 800 counties throughout the 50 US states and territories, the existing infrastructure is present to support expansion of this pediatric obesity prevention model serving limited-resource families in urban and rural settings.^51,52^

## Strengths and Limitations

A strength of this study was that multiple approaches to investigate feasibility were employed and included both quantitative and qualitative methods. And that the intervention content was designed utilizing existing evidence-based curricula.^23,26,27,27,29,30^ Study limitations include the use of a 1-group pre/posttest design to test limited efficacy feasibility. Parent participants were predominately female which is not uncommon in group-based community nutrition interventions but further exploration is needed to encourage male caregiver participation. The lack of random selection from a broader audience of low-income adults limits the generalizability of these results and hence their external validity. Lack of randomization to treatment and control groups limit the internal validity of the results although testing the intervention effectiveness was beyond the scope of the study.

## Next Steps

This strategy is feasible in our study with 5 clinics and next steps should include a larger sample of clinics with a focus on how to effectively recruit clinics. A recruitment and implementation manual should be developed. Assessments were based on self-report data only and shared method variance may be a factor. As such, next steps should also include objective measures such as child blood biomarker and anthropometrics measures. A 6-month follow-up data collection period should be added to assess retention of behavior and knowledge change. Lastly, further investigation is needed to optimizing referrals and clinic integration processes.

## Conclusions and Implications

The approach’s accessibility and economy of scale has the potential to serve thousands of English and Spanish-speaking, limited-resource families participating in federal programs and expand the reach of EFNEP into medical clinics in areas and states where interest exists in scaling up. Physicians and referred parents responded favorably to the EFNEP intervention in a medical clinic setting in this small study. The feasibility of continuing and replicating this pediatric clinic-EFNEP partnership model is dependent on physician and medical staff support. Challenges include accumulating the necessary quantity of referrals to fill parent classes and EFNEP staff time required for parent enrollment. The proposed model for collaboration has the potential to expand the reach of EFNEP into medical clinics and for medical clinics to offer more pediatric obesity prevention services.

## Supplemental Material

sj-docx-1-jpc-10.1177_21501327211009695 – Supplemental material for Feasibility of Colocating a Nutrition Education Program into a Medical Clinic Setting to Facilitate Pediatric Obesity Prevention Click here for additional data file. Supplemental material, sj-docx-1-jpc-10.1177_21501327211009695 for Feasibility of Colocating a Nutrition Education Program into a Medical Clinic Setting to Facilitate Pediatric Obesity Prevention by Mical K. Shilts, L. Karina Diaz Rios, Katherine H. Panarella, Dennis M. Styne, Louise L. Lanoue, Christiana M. Drake, Lenna Ontai and Marilyn S. Townsend in Journal of Primary Care & Community Health
