# Supplementary material for: Feasibility of Colocating a Nutrition Education Program into a Medical Clinic Setting to Facilitate Pediatric Obesity Prevention
Source: J Prim Care Community Health. 2021 Apr 13;12:21501327211009695. doi: 10.1177/21501327211009695 (PMC8047825; doi:10.1177/21501327211009695)
Supplement: sj-docx-1-jpc-10.1177_21501327211009695 – Supplemental material for Feasibility of Colocating a Nutrition Education Program into a Medical Clinic Setting to Facilitate Pediatric Obesity Prevention [file sj-docx-1-jpc-10.1177_21501327211009695.docx]

**Supplementary Materials**

| **Table S1.** Expanded Food and Nutrition Education Augmented Pediatric Obesity Prevention Medical Clinic Intervention Content | | |
| --- | --- | --- |
| **Week** | **Traditional EFNEP Content from Eating Smart Being Active Curriculum** | **Added Content to Support Pediatric Obesity Prevention** |
| 1 | Physical activity promotion with focus on family oriented activities. | Pediatric obesity risk assessment tools focusing on current nutrition, physical activity and child feeding practices to tailor Guided Goal Setting (GGS)  Motivational Modeling (MM)-Greatest Show on Earth Emotion-based message and facilitated conversation on the importance of parents being a role model of health behaviors at home. |
| 2 | Assess family fruit and vegetable consumption and promote variety. | Tailored GGS printout. Parents receive basic goal setting instruction on major and minor goals and attributes known to enhance goal attainment: keeping goal content positive, specific, proximal, and attainable.  Participants are guided to review the two major goals offered and select one. They select a minor goal for the week while having the option to create their own minor goal. Parents complete a goal contract.  Goal reminder cards go home to a prominent location so other family members can be aware of the goal.  My Healthy Plate (MHP) Introduction to plate proportion recommendations and child placemat sent home for parent/child activity. |
| 3 | Practice meal planning and label reading. | GGS goal effort and attainment reported on a graphically appealing tracking sheet. Parents review current goals. They can choose to continue working on the same goal, select a new goal from a preformatted list, alter the existing goal, or create their own goal.  Parents are asked to identify specific barriers to their selected goals. As a group, they develop barrier solutions which are recorded in the participant workbook (Prompt barrier identification).  My Healthy Plate (MHP) meal planning activity. |
| 4 | Identify benefits and options of whole grain foods. | GGS goal effort and attainment reported on a graphically appealing tracking sheet. Parents review current goals. They can choose to continue working on the same goal, select a new goal from a preformatted list, alter the existing goal, or create their own goal.  MM-Perseverance & modeling goal setting  Story and facilitated conversation on persevering when working towards goals.  HHF: Begin Healthy Habits Early  Introduces parents as children’s first and most important teacher. Outlines the roles parents and children assume in the feeding dynamic. |
| 5 | Promote behaviors associated with building strong bones. | GGS goal effort and attainment reported on a graphically appealing tracking sheet. Parents review current goals. They can choose to continue working on the same goal, select a new goal from a preformatted list, alter the existing goal, or create their own goal.  Parents write down three ways to reward themselves for reaching their goals.  HHK: Trying New Foods  Teaches about the developmental importance of familiarity in the acceptance of new things. Gives tips for how to introduce new foods to build familiarity and exploration. |
| 6 | Explore lean protein sources of protein and food safety tips. | GGS goal effort and attainment reported on a graphically appealing tracking sheet. Parents review current goals. They can choose to continue working on the same goal, select a new goal from a preformatted list, alter the existing goal, or create their own goal.  MM: Long term impact on kids  Story and facilitated conversation on the long-term health consequences of everyday actions, such as offering healthy meals to children. Emotion-based message on ways to grow happy kids by creating a healthy environment at home.  HHF: Cooking with Kids  Stresses the importance of hands-on exploration for learning and gives tips for how to involve children in food preparation.  MHP activity comparing adult and child recommended food group portion sizes. |
| 7 | Select foods low in fat, sugar, and salt. | GGS goal effort and attainment reported on a graphically appealing tracking sheet. Parents review current goals. They can choose to continue working on the same goal, select a new goal from a preformatted list, alter the existing goal, or create their own goal.  Small group activities integrated throughout the intervention to facilitate discussion of goal effort, attainment, barriers, and cues. These discussions are facilitated by the educator to promote social comparison and social support.  MM: Powerful family meals  Emotion-based message on the importance of family meals to create strong family connections and support physical and emotional health. |
| 8 | Review of key concepts and discuss involving family in new healthy behaviors. | GGS goal effort and attainment reported on a graphically appealing tracking sheet.  Parents are guided to discuss why some goals are not accomplished or are hard to sustain. Techniques are provided to promote successful goal development, attainment, and sustainability. Participants are guided to develop their own goal to work on after the intervention has concluded.  MM: Life lasting gifts  Emotion-based message on the importance of family meals to create strong family connections and support physical and emotional health. |

| **Table S2.** Question Guide for Parent Interviews Post Intervention |
| --- |
| The people who designed these classes are very interested in knowing your opinion…  *Las personas que diseñaron estas clases están muy interesadas en conocer su opinión...* |
| 1. In general, how did you like these classes?   *En general, ¿qué le parecieron las clases?* |
| 1. Was there something you found particularly useful about these classes?   *¿Hubo algo acerca de estas clases que le haya sido especialmente útil?* |
| 1. Was there anything you think should be removed from the classes because it was not useful?   *¿Hubo algo que considera se debería quitar de las clases porque no es tan útil?* |
| 1. Do you have any suggestions to make these classes better?   *¿Tiene alguna sugerencia sobre de cómo mejorar estas clases?*  4a. What do you think about the location and the room where the classes were held?  *¿Qué le parecieron la ubicación y el salón donde se dieron las clases?*  4b. What do you think about the day and time of the day when the classes were offered?  *¿Qué le parecieron el día y la hora a la que se dieron las clases?*  4c. What do you think about the length and number of classes offered?  *¿Qué le parecieron la duración y el número de clases que se ofrecieron las clases?* |
|  |

| **Table S3.** Parent 8-item Self-Administered Medical Clinic Community Nutrition Intervention Feasibility Survey |
| --- |
| 1. Clinic name. |
| 1. Tell us why you came to the classes.    1. Class topics    2. Doctor referral    3. Classes in my medical clinic    4. Stipend    5. other |
| 1. How did you find out about the classes? |
| 1. What about the timing of the classes? |
| 1. Tell us how you liked these.    1. Food Tastings    2. Goal Setting    3. Parenting Topics    4. Nutrition Topics    5. Motivational Messages & Stories    6. other |
| 1. Do you think other parents you know would attend these classes? |
| 1. What might prevent other parents you know from coming to these classes? |
| 1. If you missed classes, tell us why? |

| **Table S4.** Physician 13-item Online Medical Clinic Community Nutrition Intervention Feasibility Survey Questions |
| --- |
| 1. Please select the clinic with which you are affiliated. |
| 1. Please select the position that describes your primary role in the clinic. |
| 1. Did you make patient referrals to the Healthy Kids Nutrition Classes using these Rx pads? |
| 1. Please select how you made patient referrals |
| 1. Please select how many referrals you made |
| 1. Please share any success stories of patients that have participated in our program |
| 1. Please indicate which statements describe why you did not make patient referrals. |
| 1. Do you think doctors at other clinics would participate in referring patients to nutrition classes? |
| 1. Comments about why other doctors may or may not refer patients |
| 1. Please list any suggestions you have to improve the Referral process (Rx pads and referral drop box) |
| 1. Please list any suggestions you have to improve the physician training and transmittal of information about the nutrition classes |
| 1. Overall, how useful would you rate the Healthy Kids/Niños Sanos project is for doctors? |
| 1. Overall, how useful would you rate the Healthy Kids/Niños Sanos project is for patients? |

| **Table S5.** University of California Davis Healthy Kids Pediatric Obesity Risk Assessment Tool (19-Items)* |
| --- |
| 1. I sit and eat a meal with my child. 2. My child eats fruit. 3. My child goes to bed around _____ P.M. |
| 1. My child eats _____ vegetables at his main meal. 2. I keep fruit ready for my child to eat. |
| 1. My child drinks milk _____ times a day. |
| 1. My child likes to play inside instead of watching TV. 2. I buy vegetables. 3. My child plays video or computer games _____ hours a day. 4. My child watches TV _____ hours a day. 5. My child eats snack foods like apples, bananas or carrots. 6. My child drinks soda or sugared drinks. 7. I buy fruits. 8. My child eats candy, cake or cookies _____ times a day. 9. My child drinks sports drinks or sugared drinks ______ times a day. 10. My child eats chips _____ times a day. 11. My child eats more than one kind of vegetable a day. 12. I trim fat before eating meat. 13. 19. My child drinks milk. |
| *Available for download at <https://townsendlab.ucdavis.edu/evaluation-research-tools/validation-research/> |
| **Table S6.** University of California Davis My Child at Mealtime Pediatric Obesity Risk Assessment Tool* |
|  |
| 1. I get my child to eat by explaining that the food is good for him. |
| 1. My child sits and eats with an adult. |
| 1. I tell my child she will get a treat for eating. |
| 1. I plan meals. |
| 1. I ask my child to try a little bit of a new food. |
| 1. I remind my child to keep eating her food. |
|  |
| 1. I prepare at least one food that I know my child will eat. |
| 1. I praise my child for eating. |
| 1. I help my child with eating (cut food, cool the food). |
| 1. I get my child to eat by making food fun. |
| 1. I tell my child he will get in trouble for not eating (no toys, time out). |
| 1. My child eats a snack at about the same time every day. |
| 1. My child eats dinner at about the same time every day. |
| 1. I struggle with my child to get her to eat (pick her up and put her in the chair). |
| 1. I warn my child he will not get a treat if he does not eat. |
| 1. I say good things about the food my child is eating. |
| 1. I ask my child to pick from foods already cooked. |
| 1. I hand-feed my child to get her to eat. |
| 1. I say to my child, “Hurry up and eat your food”. |
| 1. I tell my child that she needs to eat an item on her plate (Eat your chicken”). |
| 1. I tell my child I do not like it that he is not eating. |
| 1. I ask my child questions about the food she is eating. |
| 1. I let my child serve himself |
| 1. I tell my child that I will reward her for eating with TV, playtime, or videogames. |
|  |
| 1. A TV is on when my child eats. |
| 1. My child skips meals. |
| 1. I beg my child to eat his food |

*Available for download at <https://townsendlab.ucdavis.edu/evaluation-research-tools/validation-research/>

| **Table S7.** California EFNEP Checklist items* |
| --- |
| 1. I plan meals. 2. I compare prices. |
| 1. I run out of food before the end of the month. 2. I shop with a list. |
| 1. Meat and Dairy. I let them sit out for more than 2 hours. 2. I thaw frozen foods at room temperature. 3. I choose healthy foods for my family. 4. I make foods without adding salt. 5. I use this food label. 6. My child eats food within 2 hours of waking up. 7. My child drinks soda __ times a day. 8. My child drinks sports or sugared drinks __times a day. 9. My child eats fast food __ times a day. 10. My child watches TV __ hours a day. 11. My child plays video or computer games. |

*Available for download at <https://townsendlab.ucdavis.edu/evaluation-research-tools/efnep-evaluation-tools-guides/>
